# An Unusual Case of Abdominal Leiomyoma Presenting as a Free Lying Intraperitoneal Mass in an Elderly Gentleman

**DOI:** 10.1155/2016/1714958

**Published:** 2016-09-08

**Authors:** Amrit Nasta, Kunal Nandy, Yogesh Bansod

**Affiliations:** Department of General Surgery, Seth G.S. Medical College and KEM Hospital, Mumbai, India

## Abstract

*Introduction*. Leiomyomas are common benign tumours of female reproductive tract and are rarely seen in extrauterine location.* Case Report*. We report an interesting case of a free lying abdominal leiomyoma presenting as a painless abdominal lump in an elderly gentleman.* Discussion*. Primary abdominal leiomyomas are uncommon and require surgical removal if symptomatic.

## 1. Introduction

Leiomyomas are the commonest benign tumour of the female reproductive tract and are found in 20% of women of reproductive age. Extrauterine leiomyomas are rare and usually benign and cause diagnostic dilemmas as they may mimic a malignancy [1]. Leiomyomas originate from smooth muscle cells of the uterus and rarely from stomach, bowel, or vessel wall. The transformation of such cells to leiomyoma involves somatic mutation and perhaps some unknown synergistic action of hormones, deranged lipid metabolism, and local growth factors [2]. Sometimes, unusual growth patterns may be seen, which include benign metastasizing leiomyoma, disseminated peritoneal leiomyomatosis, intravenous leiomyomatosis, parasitic leiomyoma, and retroperitoneal masses. In the presence of such patterns, a synchronous uterine leiomyoma or a previous surgery for removal of a primary uterine tumour may be indicative of the diagnosis [3]. We report an unusual free lying intraperitoneal leiomyoma presenting as an abdominal lump in a gentleman.

## 2. Case Report

**Figure 1:**
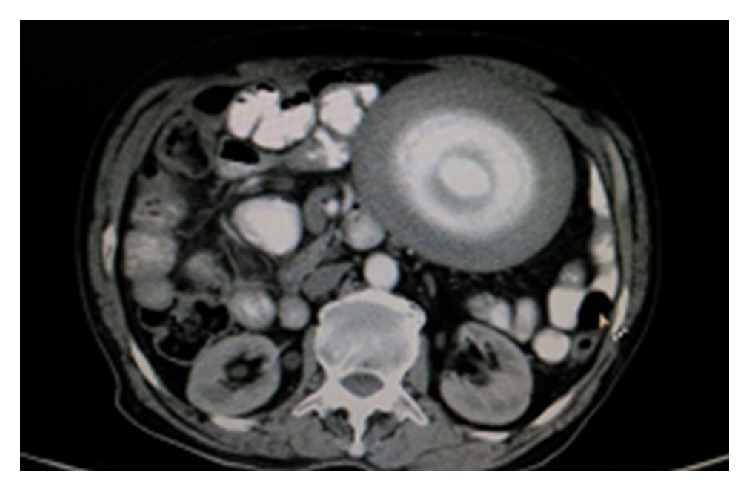
Contrast CT abdomen showing concentrically calcified mass.

**Figure 2:**
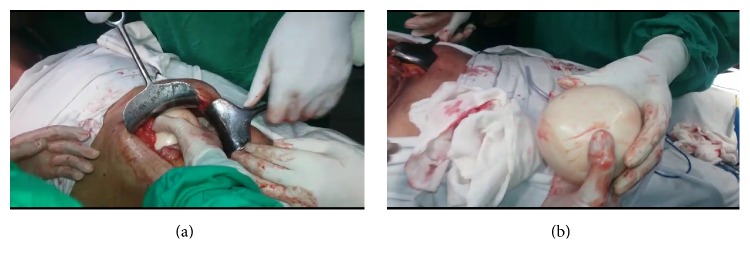
Intraoperative appearance of tumour.

**Figure 3:**
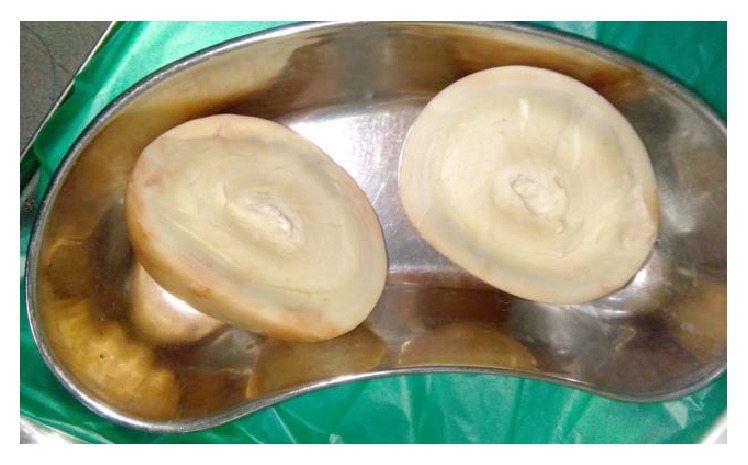
Cut open specimen of tumour.

**Figure 4:**
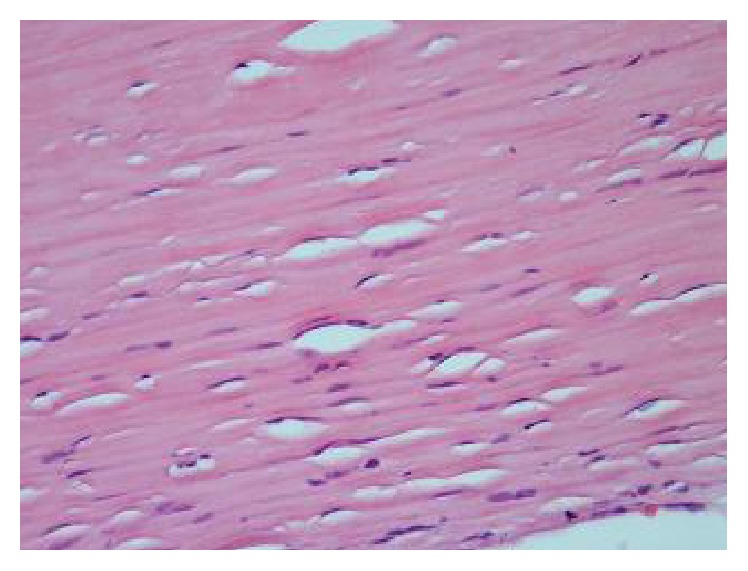
Histopathology (H&E stain) showing paucicellular, unencapsulated hyalinised tissue with calcification.

**Figure 5:**
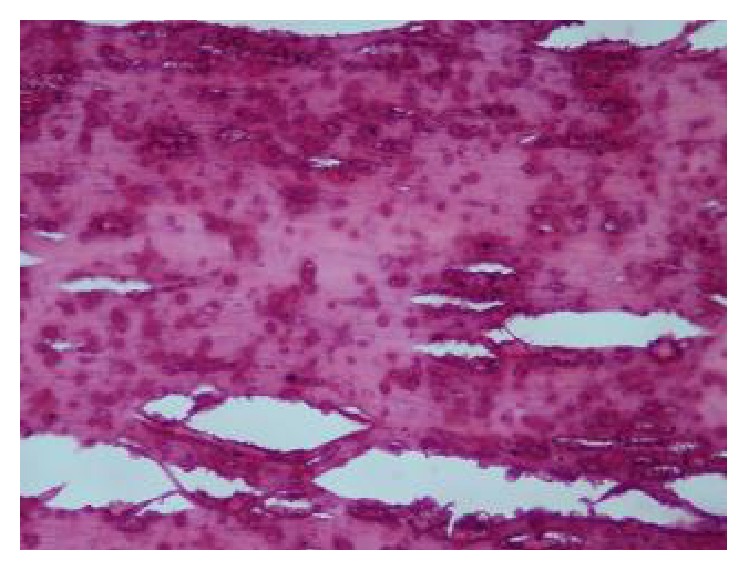
Histopathology slide showing stretched out nuclei at periphery without atypia.

A 72-year-old male patient with no comorbid illnesses came with chief complaints of constipation since 1 year and left upper abdominal lump since 3-4 months. There was no history of bleeding per rectum, vomiting, or loss of weight. He had no previous surgical history. On examination there was a palpable lump of 9 × 9 cms in the left hypochondrium extending into the epigastrium and left flank. Lump was hard and surface was smooth, showing minimal mobility and upper border could not be defined. Patient had undergone a colonoscopy elsewhere which was normal. A contrast CT abdomen was done which showed a concentric calcified mass in the left hypochondrium, free from bowel, reported as a calcified mesenteric cyst (Figure 1). Patient was posted for surgery in light of the symptoms and radiology findings. An exploratory laparotomy has been done which showed a free lying, hard, intraperitoneal mass of 10 × 12 cm size in the left hypochondriac region (Figure 2). Mass was free from bowel, mesentery, and peritoneum. It showed no vascularity or attachments and could be retrieved with minimal dissection. Entire abdominal cavity was examined for presence of any other similar lesion or origin of tumour. Abdomen was closed in layers. Postprocedure patient was started on liquids after 8 hours which he tolerated well and discharged on postoperative day 1. Specimen showed whorled appearance on cut section (Figure 3). Specimen was sent for histopathology which showed paucicellular, unencapsulated, hyalinised tissue with calcification, along with stretched out cells at periphery without atypia suggestive of leiomyoma (Figures 4 and 5). Immunohistochemistry was inconclusive in view of paucicellularity. Slide block was sent to another pathologist for second opinion who also opined in view of leiomyoma but confirmation could not be made in view of lack of cellularity. Patient was asymptomatic on follow-up after 6 months.

## 3. Discussion

Extrauterine leiomyoma is a rare entity. Disseminated peritoneal leiomyomatosis and parasitic leiomyoma are the variants of extrauterine leiomyoma that are found within the peritoneal cavity. A primary peritoneal leiomyoma was reported by Singh et al. in a 51-year-old woman [4]. Parasitic leiomyomas have been reported in the abdominal wall and retroperitoneum. It has been suggested that the uterine leiomyoma becomes adherent to these structures, develops its own blood supply from them, and gradually loses its attachment with the uterus, thus developing as a parasite at the new location [5]. Somatic soft tissue leiomyomas commonly present as large localized masses. Macroscopically, they present as well circumscribed mass which are usually surrounded by a fibrous pseudo-capsule. Histologically, they lack atypia and necrosis and are mitotically inactive (<1 mitosis/50 high power fields) [6]. The retroperitoneal leiomyomas share a histologic similarity to uterine leiomyomas. Malignant leiomyosarcomas occur more commonly in the retroperitoneum, followed by deep somatic soft tissue, and are diagnosed by the presence of nuclear atypia and essentially any level of mitotic activity. Leiomyosarcomas of deep somatic tissue usually arise from small veins [7]. Immunohistochemical staining for desmin and actin, markers of smooth muscle differentiation, can be performed to detect these markers in leiomyomas [8]. Leiomyomas are benign tumours and their surgical removal is curative. In our case, the tumour was lying freely in the peritoneal cavity, which has never been reported before in literature. With this being a male patient, the possibility of a parasitic leiomyoma is ruled out. It is possible that the tumour arose primarily from bowel or retroperitoneum as exophytic growth, which got disconnected and underwent calcific change leading to a free lying intraperitoneal tumour. It presented a diagnostic dilemma to the surgeon as well as the radiologist, requiring pathological confirmation.

## 4. Conclusion

Primary leiomyoma of the abdominal cavity is an unusual entity and surgical removal of the tumour is curative.
